# Increased Vulnerability to Dehydration Due to Heat Stress and Drought Across Reproductive States for Pastoralist Women in Northern Kenya

**DOI:** 10.1002/ajhb.70303

**Published:** 2026-06-30

**Authors:** Suha Arshad, Kedir T. Roba, Natalie Meriwether, Hannah Jacobson, Amanda McGrosky, Anna Tavormina, Nicole Bobbie, Grace Khosi, Matthew Douglass, David R. Braun, Rosemary Nzunza, Emmanuel Ndiema, Herman Pontzer, Asher Y. Rosinger

**Affiliations:** ^1^ Department of Anthropology Vanderbilt University Nashville Tennessee USA; ^2^ Department of Biobehavioral Health Pennsylvania State University University Park Pennsylvania USA; ^3^ Hubert Department of Global Health Rollins School of Public Health Atlanta Georgia USA; ^4^ Department of Anthropology Northwestern University Evanston Illinois USA; ^5^ Department of Biology Elon University Elon North Carolina USA; ^6^ Global Health Institute, Duke University Durham North Carolina USA; ^7^ Department of Earth Sciences National Museums of Kenya Nairobi Kenya; ^8^ College of Agricultural Sciences and Natural Resources and Agricultural Research Division University of Nebraska‐Lincoln Lincoln Nebraska USA; ^9^ Center for the Advanced Study of Human Paleobiology The George Washington University Washington DC USA; ^10^ Center for Virus Research Kenya Medical Research Institute (KEMRI) Nairobi Kenya; ^11^ Department of Evolutionary Anthropology Duke University Durham North Carolina USA; ^12^ Department of Anthropology Pennsylvania State University University Park Pennsylvania USA

**Keywords:** drought, heat stress, hydration, lactation, pastoralist, pregnancy, reproductive status

## Abstract

**Objectives:**

Extreme weather events, like drought and heat stress, make it harder to meet water needs in water‐insecure settings, particularly vulnerable groups. This study examines how short‐term (heat stress) and long‐term (drought) water stress affects hydration status across reproductive states (pregnant, lactating, compared to non‐pregnant/non‐lactating) for Daasanach semi‐nomadic pastoralist women in northern Kenya.

**Methods:**

Drawing on unbalanced panel data, we analyzed 565 observations from 303 women (aged ≥ 16 years) in 2019–2024. Hydration was assessed via urine specific gravity (USG) with dehydration classified as USG > 1.020. Environmental heat stress was measured by ambient temperature and humidity, with sensitivity analyses using wet bulb globe temperature.

**Results:**

Mixed effect logistic regression models indicated ambient temperature and humidity were significantly associated with greater odds of dehydration across all women. Holding heat stress constant, lactating but not pregnant women had higher odds of dehydration than non‐pregnant/non‐lactating women. A significant interaction between heat stress and reproductive status indicated that the probability of dehydration increased fastest for pregnant women as temperatures rose. Holding heat constant, dehydration probability increased during drought years compared to pre‐ and post‐drought and was most pronounced among lactating women.

**Conclusions:**

Ambient heat stress increases dehydration risk among Daasanach women with effects compounded in pregnancy, though overall lactation was the period of greatest vulnerability to dehydration. Dehydration probability peaked during the drought illustrating how long‐term periods of water scarcity also challenge water needs. Heat stress and droughts exacerbate maternal and infant health risks; thus, targeted hydration and cooling interventions are needed.

## 1 Introduction

Hotter global temperatures and temperature extremes worldwide have profound consequences for human health and well‐being (Luber and McGeehin 2008; Rosinger 2023). In addition to increased global maximum and average daily temperatures, research has shown that climate change has amplified the frequency and severity of droughts, flooding, dust storms, and other extreme weather events (Romanello et al. 2024). The World Health Organization (WHO) estimates that climate change is already responsible for an estimated 37% of recent heat‐related deaths and is projected to drive an additional 250 000 deaths each year from malnutrition, malaria, diarrhea, and heat stress from 2030–2050 (World Health Organization 2023). The burden of climate‐related morbidity and mortality is disproportionately borne by residents of low‐ and middle‐income countries as well as vulnerable groups (United Nations Children's Fund 2023).

Pregnant and lactating individuals are particularly at risk for heat stress due to the physiological demands of reproduction. During pregnancy, plasma volume expands by approximately 30%–50% (Vricella 2017), glomerular filtration rate increases (Cheung and Lafayette 2013), cardiac output increases, and metabolic rate rises to meet the demands of fetal growth (Costantine 2014). Although pregnancy is accompanied by physiological adaptations that enhance heat dissipation, extreme heat exposure combined with increased body mass during pregnancy can impair the body's ability to shed excess heat (Bonell et al. 2020; Samuels et al. 2022). The physiological demands of reproduction continue during lactation. Breastmilk is 88% water (Kim and Yi 2020), resulting in roughly 400–800 mL of maternal water loss per day during lactation depending on milk volume produced (Neville et al. 1988). These physiological changes substantially raise water needs making it harder to meet said needs. Water turnover (liters/day) increases ~20% in the 3rd trimester and ~8% during lactation in industrialized populations (Yamada et al. 2022). Daily water intake recommendations are 2.7 L for non‐pregnant/non‐lactating women, increase to 3.0 L for pregnant women, and further increase to 3.8 L for lactating women (Food and Nutrition Board of the Institute of Medicine 2004). Water scarcity that occurs during droughts likely exacerbates this heat stress making it harder to meet water needs.

Overall, extreme heat places increased strain on human thermoregulation and hydration, with heat stress referring to the inability of the body to dissipate heat and maintain internal homeostasis (Rosinger 2023). Prolonged exposure to high temperatures can lead to heat‐related illness and mortality once environmental conditions surpass the body's capacity for heat dissipation (Vecellio et al. 2022). Sweating, a primary mechanism of thermoregulation, allows for heat dissipation but also increases water loss, raising dehydration risk and overall water needs. Negative health effects of long‐term heat exposure include cardiovascular disease, heat‐derived lung damage, and chronic kidney disease (Ebi et al. 2021).

These risks may be especially consequential during reproduction. Exposure to extreme heat while pregnant has impacts for maternal and fetal health. Heat exposure in the first trimester has been linked to increased hospitalizations for early pregnancy hemorrhage, antepartum bleeding, early labor, and hyperemesis, and is associated with reduced birth weight in offspring (Kim et al. 2019; Chersich et al. 2020). In the final weeks of pregnancy, high ambient temperatures have been shown to increase the risk of preterm birth and stillbirth (Chersich et al. 2020). Hypertensive disorders of pregnancy have also been linked to heat exposure (Mao et al. 2023). Heat stress may also influence infant nutrition, as hormonal changes due to heat stress, namely the production of catecholamines and elevated cortisol, impact breastmilk production and composition (Traylor et al. 2024).

The risks of heat stress are intensified when extreme heat occurs alongside drought. Drought conditions increase the likelihood and intensity of extreme heat events while also constraining access to water. As soil and vegetation dessicate with drought, deficits in vapor pressure reduce evapotranspiration and raise near surface air temperature, a mechanism referred to as soil moisture‐temperature feedback (Vogel et al. 2017). Soil moisture‐temperature coupling can amplify heat extremes beyond the changes in climate alone (Zhou et al. 2019). Simultaneously, decreased rainfall during drought lowers surface water levels and prevents underground aquifers from being replenished, forcing households to make longer and more frequent trips to increasingly distant water sources (UNICEF 2022). While hydration traditionally serves as a protective mechanism against heat strain, drought constrains water access and consumption patterns. Communities facing chronic water insecurity are at increased risk of adverse health outcomes due to hypohydration (Bethancourt et al. 2021). In this way, drought exacerbates vulnerability to the effects of extreme heat by simultaneously increasing thermal load and constraining hydration.

These intersecting pressures are particularly important in pastoralist settings, where livelihoods depend heavily on local environmental conditions and access to water. Populations living in parts of Africa and Asia are currently exposed to some of the world's highest extreme temperature frequencies and intensities (Moyo et al. 2023). From October 2020 to April 2023, the Greater Horn of Africa experienced five consecutive failed rainy seasons, culminating in the region's most severe drought in 40 years (Roba et al. 2025). Widespread crop failures, livestock losses, and displacement intensified food and water insecurity for approximately 20 million people (Odongo et al. 2025). Households reliant on subsistence‐based livelihoods faced heightened exposure to extreme heat under these conditions. Pastoralist communities, who depend on livestock and livestock byproducts as a source of food and income, are particularly vulnerable to the effects of drought due to the combined pressures of heat stress and water scarcity, with documented impacts on their physical health and psychosocial wellbeing (Tofu et al. 2025; Rosinger et al. 2024).

Previous studies note how environmental stressors are challenging long‐standing human adaptive strategies, particularly with the physiological trade‐offs made during reproduction, and call for better understanding of how women adapt to the overlapping burdens of reproductive physiology, heat stress, and water insecurity (Howells et al. 2025; Bhandari et al. 2025). Prior cross‐sectional work with Daasanach women demonstrated that greater heat stress was associated with elevated odds of dehydration. Risk of dehydration (while elevated) was not significantly greater among both pregnant and lactating women potentially because of sample size (Bethancourt et al. 2021). Cross‐sectional analyses are limited in their ability to infer change over time or test how water needs change during prolonged water stress events (Bethancourt et al. 2021). Examining these dimensions longitudinally, especially over the course of extreme water stress such as a drought, allows for better understanding of how women's physiological responses to heat vary across changes in their reproductive stages, time, and environmental conditions outside of heat exposure alone. These factors are critical to understand as pregnant and lactating women are vulnerable to dehydration and heat stress.

The overarching question this study aims to test is how short‐term and long‐term water stress events affect dehydration risk for women across reproductive states. Therefore, this study investigates the relationship between environmental heat stress, drought, and hydration status across reproductive states (pregnancy, lactation, or non‐pregnant/non‐lactating) among Daasanach semi‐nomadic pastoralist women from before to after the historic 2020–2023 drought. Building on previous work examining hydration status among lactating and pregnant women living in hot environments (Rosinger 2015; Bethancourt et al. 2021), this unbalanced panel study incorporates both acute heat stress and a drought event by studying the relationship over a five‐year span. This paper has two linked aims. First, it examines how ambient heat stress (ambient temperature alongside humidity) as a proxy for short‐term water stress is associated with hydration status across reproductive states, and how heat stress interacts with reproductive states to affect their dehydration risk. Second, it assesses how the probability of dehydration changes for women by reproductive state over the course of the historic drought as a long‐term water stress event that changes water availability and increased water insecurity.

## 2 Materials and Methods

### 2.1 Study Population

Daasanach semi‐nomadic pastoralists are a population indigenous to northern Kenya and southwestern Ethiopia, with estimated populations of approximately 19 000 and 48 000, respectively (Kenya National Bureau of Statistics (KNBS) 2019; Mwamidi et al. 2018). Communities live near Lake Turkana, an alkaline, moderately saline desert lake, in a hot, semi‐arid environment. Average annual daytime temperature is 30.3°C making it one of the hottest places on earth (Weather and Climate 2020). The climate follows a bimodal rainfall pattern with two rainy seasons, March–May and October–December, and two dry seasons, May–October and January–February (Ojwang et al. 2018).

Data for this study were collected during the early dry seasons with characteristically low rainfall and chronic water insecurity, conditions that have intensified in recent years due to a prolonged regional drought in the Horn of Africa. Though Ethiopian Daasanach engage in flood‐retreat cultivation, Daasanach in northern Kenya rely on livestock herding and fishing (Wall 2020). Male family members are traditionally responsible for herding livestock while Daasanach women are often responsible for water and firewood collection, childcare, homebuilding/maintenance, and cooking (Sadhir et al. 2025).

### 2.2 Daasanach Women and Water Needs

Daasanach women experience unique hydration challenges under environmental heat stress due to the combined pressures of physiological demands and labor responsibilities. Daily water turnover refers to the total volume of water consumed and lost by the body each day, and is typically measured in liters (Yamada et al. 2022). Among Daasanach women, absolute water turnover has been measured at 7.46 L/day and found to be significantly higher than other comparable subsistence‐based communities and higher than global water turnover averages for women of 3.4 L/day (McGrosky et al. 2024, 2025). Water turnover accounts for water lost through sweating, respiration, and physical activity, with higher turnover rates requiring proportionally higher water intake to maintain hydration. Previous research has documented high water insecurity among Daasanach communities, pointing toward a mismatch between their high physiological water requirements and limited access to safe and reliable water sources (Bethancourt et al. 2023; Ford et al. 2023; Rosinger et al. 2024; Roba et al. 2025).

Water insecurity in Daasanach communities is often mitigated through water borrowing between households. Individuals with greater social connectivity to water‐sharing networks experience lower levels of water‐insecurity related perceived stress (Ford et al. 2023). Among women, especially those who are pregnant, these networks may be particularly protective. Pregnancy can prompt shifts in household labor dynamics, with increased community and family support for tasks like water collection and firewood gathering (Sadhir et al. 2025). Behavioral accommodations during pregnancy include increased water consumption and reduced physical exertion over time as women receive more assistance with physically demanding tasks (Sadhir et al. 2025).

Breastfeeding practices among Daasanach women generally align with World Health Organization (WHO) recommendations for exclusive breastfeeding during the first 6 months of life (World Health Organization 2011). Survey data from the study population in 2024 indicate a mean duration of 5.8 months of exclusive breastfeeding, with continued breastfeeding commonly extending to 2 years and beyond. Seasonal variations in labor demands and heat exposure may exacerbate the hydration burden experienced by Daasanach women in this context. The dry season is a period marked by increased environmental heat stress and greater time spent collecting water (Sadhir et al. 2025).

### 2.3 Ethical Approval and Data Collection

This study is part of the Daasanach Human Biology Project, a longitudinal study designed to investigate Daasanach lifestyle and health in response to environmental stressors, sociocultural changes, and food and water insecurity. All data were collected in accordance with procedures approved by the Pennsylvania State University's Institutional Review Board (STUDY00009589), the Kenya Medical Research Institute (KEMRI/SERU/CVR/003/3739), the Marsabit County Director of Health, and Daasanach community leaders, while Daasanach participants provided oral and written (fingerprint if illiterate) informed consent.

Individual‐level and weather data for this study were gathered during the dry season (June–July) in 2019, 2022, 2023, and 2024, with no data collected during 2020–2021 due to the COVID‐19 pandemic. Data were collected at the same time each year to be comparable across seasonal and environmental dimensions. Seven Daasanach communities located at various distances from the town of Illeret (4.314° N, 36.227° E) were sampled for this study, with an eighth added in 2023. Seven of the eight communities sampled were relatively permanent, and one community was nomadic. We recruited households with two household heads, male and/or female (some female household heads were as young as 14 due to cultural marriage norms), and up to two children between the ages of 4 and 16.

Participants were initially recruited in 2019 by community mobilizers and health volunteers through random sampling of every third household. The 2022–2024 surveys attempted to recontact these original participants and replaced missing households with neighboring ones when necessary due to the mobile lifestyle of the pastoralist population as well as expanded it to new community members to reflect changes in the community characteristics. As a result of either temporary or permanent attrition between study waves, this resulted in an unbalanced panel. Each participant was contacted once per field season. Household interviews, spot urine collection, and environmental heat measurements occurred on the same day for each participant, typically within a 1‐h window, to best pair environmental exposure to hydration status. For the purposes of this study, the analytic sample consisted of women aged ≥ 16 years old (as 16 was the youngest in our sample that an individual was reported as pregnant and lactating) with complete hydration and environmental exposure data. Male participants and individuals with missing USG values or reproductive status were excluded.

### 2.4 Study Variables

#### 2.4.1 Exposure: Environmental Heat Stress

Measures of environmental heat stress included ambient temperature (°C) and relative humidity, as well as supplementally wet bulb globe temperature (WBGT) (°C), which was incorporated in the study data collection in 2022. Ambient temperature refers to the outside air temperature, whereas WBGT is a composite measure that accounts for ambient temperature, relative humidity, wind speed, sun angle, and solar radiation (Vecellio et al. 2022). Human biologists use ambient temperature and WBGT as ecologically valid indicators of thermal load in dry environments as compared with heat index, especially in field settings where direct measurements of heat exposure are challenging (Baker et al. 2025). This study recorded ambient temperature and humidity using a digital Kestrel 3000 Pocket Weather Meter in 2019 and beginning in 2022 the Kestrel 5400 Weather Meter which also assesses WBGT (Kestrel; Boothwyn, PA, USA) placed in direct sunlight and unblocked from wind. Temperature data were recorded at the time of urine sample collection during daytime fieldwork hours, typically between 08:00 and 17:00, with a typical break at mid‐day (Figure S1). Data collection took place at the same times of day each field season, and each survey wave took place between mid‐June through mid‐late‐July during the early dry season. Thus, these measures reflect local environmental conditions at the time of participation.

#### 2.4.2 Exposure: Drought

Drought is treated as an environmental exposure due to the study period spanning before, during, and after a prolonged regional drought that markedly reduced water availability for Daasanach households. Drought was captured according to survey year, which aligns with observed declines in rainfall. Survey year was modeled as a series of indicator variables to capture temporal variability in climatic conditions across the drought. 2019 was the pre‐drought year and served as the reference year; 2022 and 2023 correspond with the peak and end of the drought, and 2024 corresponds with a year post‐drought.

#### 2.4.3 Primary Predictor: Reproductive Status

Reproductive status was self‐reported during household surveys. All women were asked if they were pregnant and, if so, for how many months. Separately, they were asked if they were lactating and, if so, for how many months. Women who reported being pregnant were coded as pregnant, regardless of lactation status (two women were both pregnant and lactating). Women who reported they were breastfeeding, but not pregnant, were coded as lactating. Women who reported they were neither pregnant nor lactating were coded as non‐pregnant, non‐lactating (NP/NL). In the analysis, we analyzed this as a categorical variable with NP/NL women used as the reference group.

#### 2.4.4 Outcome: Hydration Status

Urine specific gravity (USG) was used as a biomarker of hydration status. USG is the best field‐friendly measure of hydration status and compares the density of solutes in urine to water and increases in response to fluid loss or inadequate fluid intake (Rosinger 2015; Wutich et al. 2020). Spot urine samples were collected from participants prior to the survey, covered, and placed in the shade for at least 10 min to reach room temperature before being analyzed in the field using an Atago handheld refractometer Pen (Model 3741). Refractometers were calibrated with distilled water, and each sample reading was repeated three times to obtain average USG. USG values range from 1.000 to 1.040, with values above 1.020 classified as dehydrated (Hew‐Butler et al. 2018; Wutich et al. 2020). Analyses present USG as a dichotomous variable of dehydration (coded as 0 = not dehydrated; 1 = dehydrated) to focus on the biologically significant state of increased water needs, but we also present continuous results in supplemental materials.

#### 2.4.5 Controls

All models adjusted for age, percent body fat, household size, and year fixed effects to control for covariates that impact hydration and heat stress. Age was reported in years during household surveys and confirmed with identification cards or estimated by the research team when missing based on historical and recent events. We adjusted for participant age due to declines in thirst perception, kidney mass, glomerular filtration rate, and urinary concentrating ability observed in older adults (Li et al. 2023). Body composition was assessed using a Tanita bioelectrical impedance analysis (BIA) scale providing percent body fat as variation in lean and fat mass influences hydration and thermoregulation (Yamada et al. 2022). Finally, we adjusted for household size as greater household members can change the water availability in the house.

### 2.5 Statistical Analyses

All statistical analyses were conducted using Stata V19.0 (College Station, TX). Significance was set at alpha < 0.05. We first descriptively visualized the bivariate relationship between heat stress and USG across reproductive status with observations pooled using fractional polynomial fit scatterplots. Next, we used mixed effects regression models to account for repeated observations of individuals nested within communities and within years. Thus, this mixed effects model contains both fixed time effects associated with the year of observation and random individual effects to address potential endogeneity.

To address aim 1, we first estimated how differences in the ambient heat stress measures and reproductive status were associated with USG as a dichotomous measure of hydration status using mixed‐effects logistic regression using the full 2019–2024 data adjusted for age, percent body fat, household size, and year fixed effects. We then re‐estimated the model with a three‐way interaction term between reproductive status, ambient temperature, and humidity to assess how the relationship between heat stress and hydration status varied by reproductive state.

For aim 2, to test how dehydration changed due to the drought, we draw on the first adjusted mixed effect logistic regression model for aim 1 presented in Table 2 and conduct post‐estimation marginal standardization (Muller and MacLehose 2014). This method generates predicted probabilities from the odds ratios across the range of covariates to demonstrate changes in dehydration from before the drought, during the drought, to post‐drought. We estimate the predicted probabilities first for women overall and then stratified by reproductive status.

#### 2.5.1 Sensitivity and Robustness Analyses

First, we re‐estimated the aim 1 models using mixed effect linear regression with USG as a continuous variable. Second, we re‐estimated the aim 1 mixed effect logistic regression models using WBGT instead of ambient temperature from 2022–2024 as a separate heat stress indicator.

Next, we conducted a robustness analysis by re‐estimating the primary aim 1 models restricting the analytic sample to women who were of reproductive age (16–51 years), which was the oldest recorded age for a woman in the sample who was lactating. We do this to assess whether the results are consistent when we exclude older women post‐menopause who may not be fully comparable to younger women.

Finally, we re‐estimated the primary aim 1 models restricting the analytic sample to women who participated in at least two survey waves so that all women in the model have repeat observations.

## 3 Results

The full analytic sample without missing data consisted of 565 observations from 303 women (aged ≥ 16 years old) between 2019 and 2024. Of the 303 women, 124 participated in one survey wave, 89 women participated twice, 67 women participated in three waves, and 23 women participated in all four survey waves. Some women had missing covariate data (e.g., BIA) in some waves and those observations dropped out of analyses.

The mean age across all years was 35.2 (SD = 13.1), with 14.9% of women pregnant and 43.0% lactating at the time of data collection (Table 1). The mean number of months reported pregnant and lactating at the time of the survey was 4.4 (range: 1–9) and 10.6 (range: 1–36) months, respectively (data not shown). Average USG was 1.011 (SD = 0.009), and 19.5% of women met the threshold for dehydration.

**TABLE 1:** Descriptive characteristics of Daasanach women aged 16 years or greater participating in the Daasanach Human Biology Project in 2019–2024.

Variable	2019 (*n* = 119)	2022 (*n* = 96)	2023 (*n* = 153)	2024 (*n* = 197)	Overall (*n* = 565)
	Mean (SD or n)	Mean (SD or n)	Mean (SD or n)	Mean (SD or n)	Mean (SD or n)
Age (years)	34.1 (12.4)	35.3 (12.8)	35.5 (13.4)	35.6 (13.6)	35.2 (13.1)
Pregnant (%)	12.6 (15)	14.5 (14)	16.3 (25)	15.2 (30)	14.9 (84)
Lactating (%)	38.7 (46)	43.8 (42)	42.5 (65)	45.7 (90)	43.0 (243)
Body fat %	22.0 (7.1)	21.6 (8.2)	21.6 (6.8)	24.5 (5.6)	22.7 (6.8)
Household size	7.1 (2.7)	6.7 (2.4)	5.8 (2.8)	6.2 (2.5)	6.4 (2.7)
Urine specific gravity	1.0107 (0.009)	1.0105 (0.009)	1.0110 (0.010)	1.0112 (0.009)	1.0109 (0.009)
Dehydrated (%)	17.6 (21)	19.8 (19)	20.9 (32)	19.3 (38)	19.5 (110)
Ambient temp (°C)	32.4 (2.8)	32.6 (2.6)	32.5 (2.4)	33.2 (2.3)	32.7 (2.5)
Humidity (%)	47.4 (12.8)	36.7 (6.3)	38.3 (6.3)	40.9 (5.6)	40.8 (8.8)
WBGT (°C)	—	27.6 (2.1)	27.3 (2.2)	28.5 (1.9)	27.9 (2.1)

*Note:* The 565 observations are from 303 unique women.

Abbreviations: Dehydrated, USG > 1.020; USG, urine specific gravity; WBGT, wet‐bulb globe temperature.

We observed non‐significant, non‐linear variation in the relation between number of months lactating and number of months pregnant and USG, respectively (Figure S2A,B). There was an increase in USG early in lactation and a gradual decline. In contrast, with pregnancy there was a drop in USG from the first month to the second month and then a gradual increase with USG (Figure S2B). However, in both cases, the 95% confidence intervals overlap across the course of the reproductive stage. In regression models among lactating women where month of lactation was added as a covariate, month was not significantly associated with USG using it as a continuous variable (β of USG = 0.0001; SE = 0.0001; *p* = 0.34) or when dichotomized to those in the earlier, more intensive phase of lactation (within first 6 months vs. 6+ months β = 0.0001; SE = 0.001; *p* = 0.94). Similarly, among pregnant mothers, month of pregnancy was not associated with USG (β of USG = 0.0004; SE = 0.0005; *p* = 0.46) (full results not shown). Since there were no significant USG differences across the timing of lactation and pregnancy, we grouped women in their respective reproductive stages in all further analyses.

**FIGURE 1:**
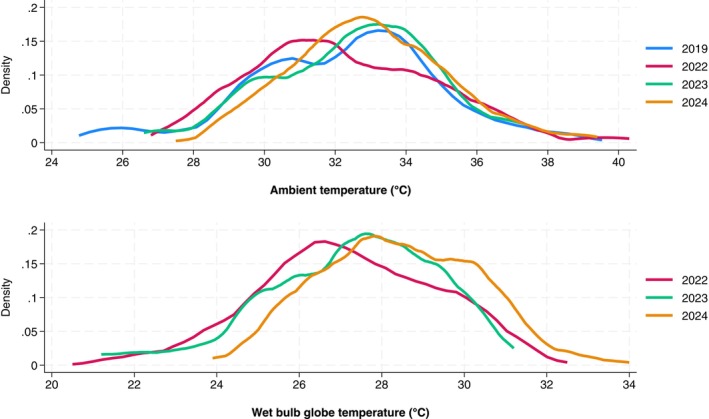
Distribution of ambient temperature and wet bulb globe temperature across study years. Ambient temperature and wet bulb globe temperature reflect heat stress at the time of urine collection during the study periods, of June–July of each year.

Environmental heat stress varied across study years during data collection (Figure 1). The pre‐drought year of 2019 had a mean ambient temperature of 32.4°C, while the 2022–2023 drought years had mean ambient temperatures of 32.6°C and 32.5°C (Table 1). In 2024, considered the first post‐drought year, the mean ambient temperature was 33.2°C (Table 1). Mean WBGT values ranged from 27.6°C in 2022 to 27.3°C in 2023 and increased to 28.5°C in 2024 (Table 1; Figure 1). Simple linear regressions found that the ambient temperature was higher in 2024 (B = 0.78°C; SE = 0.23; *p* = 0.007) than in 2019; similarly, WBGT was higher in 2024 (B = 0.96°C; SE = 0.25; *p* < 0.001) than in 2022 (full results not shown).

### 3.1 Aim 1: Heat Stress, Reproductive Status, and Dehydration

**FIGURE 2:**
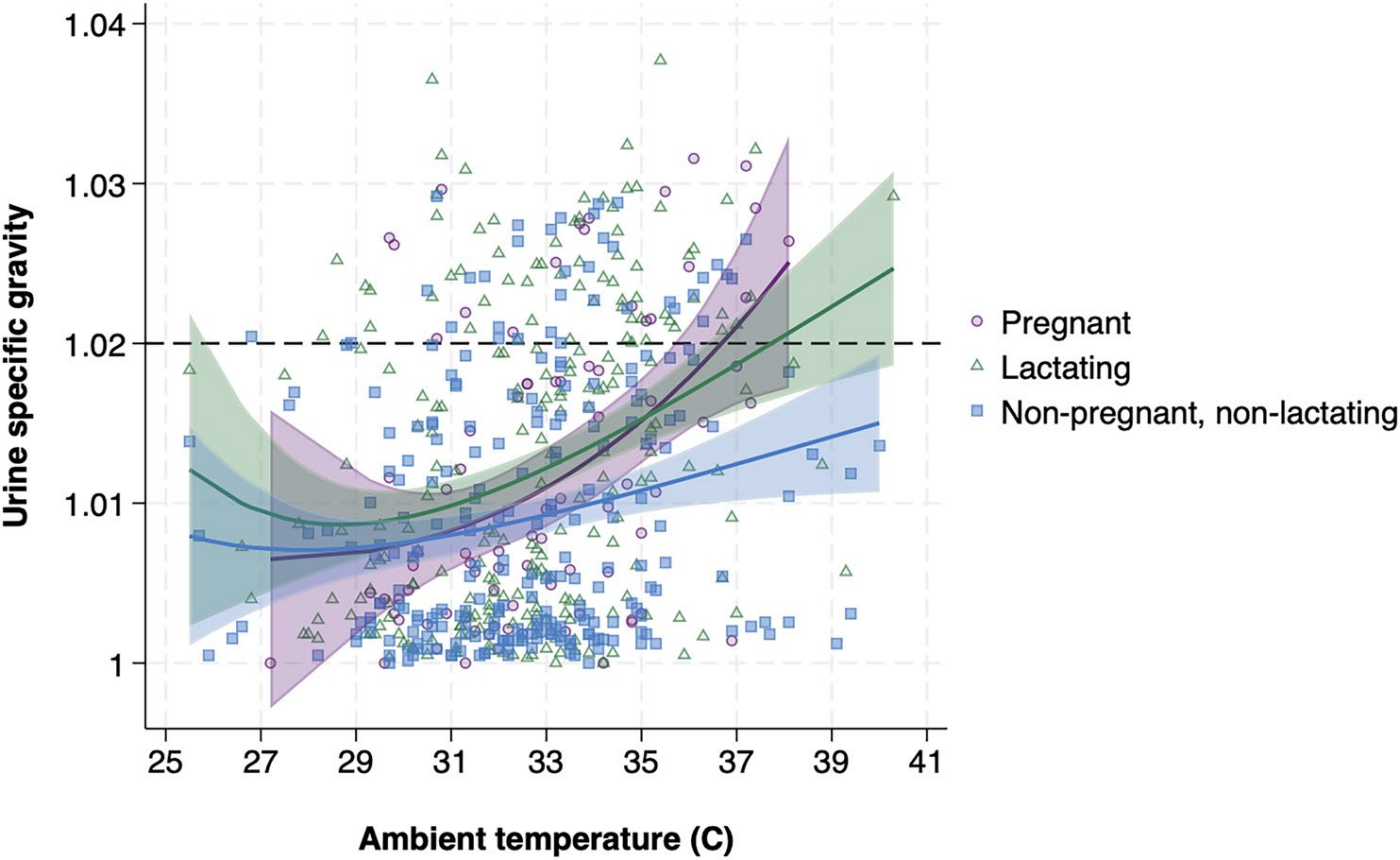
Fractional polynomial fit showing bivariate association between hydration status (USG) and ambient temperature among Daasanach women by reproductive status. The reference line at USG = 1.020 represents the cutoff used to classify dehydration.

Fractional polynomial fit scatterplots demonstrate that the bivariate relationships between ambient temperature and WBGT measures and USG appear to be partially quadratic in nature for pregnant and lactating women with a more linear relation for NP/NL women (Figure 2; Figure S3). Pregnant women appear to cross the threshold to dehydration at lower temperatures (36°C), followed by lactating women (38°C), whereas NP/NL women on average did not reach the dehydration threshold (Figure 2).

Results from the mixed effects logistic regression model show that ambient temperature and humidity at the time of urine collection were significant positive predictors of hydration status (Table 2). Each 1°C increase in ambient temperature raised the odds of dehydration by 40% (OR = 1.40, 95% CI: 1.35–1.45; *p* < 0.001), and each 1% increase in humidity raised the odds by 3% (OR = 1.03, 95% CI: 1.02–1.05; *p* < 0.001) (Table 2). Odds of dehydration also varied by reproductive status as lactating women, but not pregnant women, had significantly higher odds of dehydration than NP/NL women (OR = 2.04, 95% CI: 1.49–2.80; *p* < 0.001) (Table 2). Finally, odds of dehydration were significantly elevated during the drought in 2022 (OR = 1.45, 95% CI: 1.24–1.70; *p* < 0.001) and 2023 (OR = 1.42, 95% CI: 1.26–1.61; *p* < 0.001) compared to 2019 (Table 2).

**TABLE 2:** Mixed effect logistic regression model testing odds of being dehydrated for Daasanach women between 2019–2024.

Variable	Odds ratio (USG > 1.020)	95% CI	*p*
Ambient temperature (°C)	1.40	[1.35, 1.45]	< 0.001***
Humidity (%)	1.03	[1.02, 1.05]	< 0.001***
Non‐pregnant, non‐lactating	Reference	—	—
Pregnant	1.49	[0.84, 2.65]	0.171
Lactating	2.04	[1.49, 2.80]	< 0.001***
Body fat %	1.02	[0.97, 1.07]	0.539
Age	0.97	[0.96, 0.99]	< 0.001***
Household size	0.90	[0.82, 0.99]	0.025*
Year: 2019 (pre‐drought)	Reference	—	—
2022 (peak drought)	1.45	[1.24, 1.70]	< 0.001***
2023 (end of drought)	1.42	[1.26, 1.61]	< 0.001***
2024 (1‐year post‐drought)	0.96	[0.78, 1.18]	0.697
Constant	1.78e−06	[1.65e−07, 1.91e−05]	< 0.001***
Observations	565		
*N*	303 women		

*Note:* Mixed effect model nested observations within year and community residence.

Abbreviation: CI, confidence interval.

*** *p* < 0.001.

* *p* < 0.05.

**FIGURE 3:**
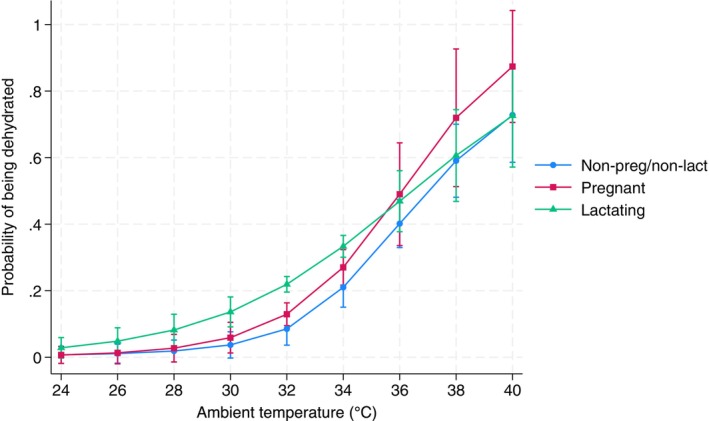
Predicted probability of dehydration by ambient temperature across reproductive states among Daasanach women. Model generated using post‐estimation marginalization to visualize interaction between heat stress and reproductive status using model presented in Table 3.

The above model was re‐estimated with a three‐way interaction term between ambient temperature, humidity, and reproductive states; several of the two‐way and three‐way interaction terms were significant (Table 3). As no term on its own is easily interpretable, we present the visualized probability of dehydration for women of different reproductive states across temperature gradients (for simplicity we visualize ambient temperature, but the estimates account for humidity in the interaction). Figure 3 demonstrates that at lower ambient temperatures, the probability of dehydration begins low for all women; for example, at 30°C, the predicted dehydration probability was 3.7% for NP/NL women, 5.9% for pregnant women, and 13.6% for lactating women. At 32°C, dehydration probabilities were 8.5%, 12.9%, and 21.9%, respectively. The probability of dehydration increased at a faster rate for pregnant women between 32°C–40°C and their probability of dehydration became highest, whereas the probability of dehydration for lactating and NP/NL women converges at the highest temperatures. For example, at 36°C, the predicted dehydration probability was 40.1% among NP/NL women, 49.0% among pregnant women, and 46.9% among lactating women. At 40°C, the predicted probability increased to 87.4% among pregnant women compared with 72.8% among NP/NL women and 72.6% among lactating women.

**TABLE 3:** Mixed effect logistic regression model testing odds of being dehydrated including three‐way interaction between heat stress and reproductive status for Daasanach women between 2019–2024.

Variable	Odds ratio (USG > 1.020)	95% CI	*p*
Ambient temperature (°C)	0.50	[0.17, 1.52]	0.223
Humidity (%)	0.40	[0.16, 0.98]	0.046*
Temp × Humidity	1.03	[1.00, 1.06]	0.032*
Non‐pregnant, non‐lactating	Reference	—	—
Pregnant	7.09e−27	[3.7e−37, 1.4e−16]	< 0.001***
Lactating	5.75e−10	[7.42e−22, 446.56]	0.128
Pregnant × Temp	5.83	[2.96, 11.47]	< 0.001***
Lactating × Temp	1.96	[0.92, 4.18]	0.081
Pregnant × Humidity	4.37	[2.15, 8.85]	< 0.001***
Lactating × Humidity	1.98	[1.03, 3.80]	0.039*
Pregnant × Temp × Humidity	0.96	[0.94, 0.98]	< 0.001***
Lactating × Temp × Humidity	0.98	[0.96, 1.00]	0.024*
Body fat %	1.02	[0.97, 1.07]	0.529
Age	0.97	[0.96, 0.99]	< 0.001***
Household size	0.90	[0.79, 1.02]	0.097
Year: 2019 (pre‐drought)	Reference	—	—
2022 (peak drought)	1.54	[1.35, 1.76]	< 0.001***
2023 (end of drought)	1.42	[1.13, 1.79]	0.003**
2024 (1‐year post‐drought)	0.96	[0.82, 1.12]	0.573
Constant	1.16e+09	[9.79e−09, 1.36e+26]	0.298
Observations	565		
*N*	303 women		

*Note:* Mixed effect model nested observations within year and community residence. Three‐way interaction includes all main terms and two‐way interactions.

*** *p* < 0.001.

** *p* < 0.01.

* *p* < 0.05.

### 3.2 Aim 2: Changes in Dehydration Probability for Women During the Drought

**FIGURE 4:**
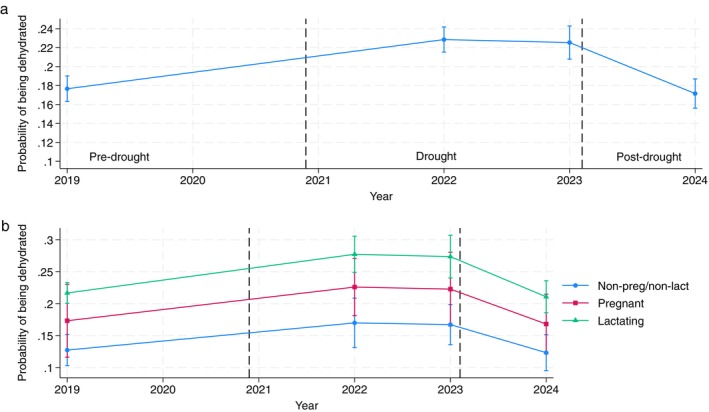
Predicted probability of dehydration in Daasanach women across pre‐drought, drought, and post‐drought periods for (A) all women and (B) by reproductive status. Dehydrated: USG > 1.020. Dashed reference lines indicate the onset and end of the historic drought, i.e., 2019 was pre‐drought, 2022 was the peak of the drought, 2023 was the end of the drought, and 2024 was 1‐year post‐drought.

Across survey years, there were temporal trends in women's probability of dehydration (Figure 4A, Table S1). Prior to the onset of the regional drought, the predicted probability of dehydration was approximately 18% (95% CI: 0.16–0.19) for women overall. This probability rose during the drought years, reaching 23% (95% CI: 0.22–0.24) in 2022 and 2023 (95% CI: 0.21–0.24). In 2024, 1 year post‐drought, dehydration probability declined to 17% (95% CI: 0.15–0.19), nearing pre‐drought levels.

The effect was more pronounced for lactating women than NP/NL and pregnant women (Figure 4B, Table S2). Among NP/NL women, the probability of dehydration increased from 13% (95% CI: 0.10–0.15) in 2019 to 17% (95% CI: 0.13–0.21) in both 2022 and 2023 (95% CI: 0.14–0.20) before declining to 12% (95% CI: 0.10–0.15) in 2024. Pregnant women had similar trajectories, with probabilities of 17% (95% CI: 0.12–0.23) in 2019, rising to 23% (95% CI 0.18–0.27) in 2022 and 22% in 2023 (95% CI: 0.17–0.28), then falling to 17% (95% CI: 0.12–0.21) in 2024. Lactating women consistently exhibited the highest dehydration risk across all years with 22% (95% CI: 0.20–0.23) predicted probability of dehydration in 2019, rising during the drought to 28% (95% CI: 0.25–0.31) in 2022 and 27% (95% CI: 0.24–0.31) in 2023, and dropping to 21% (95% CI: 0.18–0.24) 1 year post‐drought in 2024.

### 3.3 Robustness and Sensitivity Analyses

Results of the sensitivity analyses using mixed effect linear regression with continuous USG data demonstrate highly consistent results to the primary analyses (Tables S3 and S4).

Second, sensitivity analyses performed using mixed effect logistic regression with WBGT as the heat stress exposure rather than ambient temperature and humidity again found highly consistent results for the first model of aim 1 (Table S5). However, the interaction between WBGT and reproductive status was not significant, unlike the primary analyses (Table S6).

Restricting the analysis of the primary models to women of reproductive age (16–51; *n* = 276 women with 496 observations) in our sample resulted in excluding 69 observations. Results were highly consistent with the primary results (Tables S7 and S8; Figure S4).

Finally, restricting the primary models to women who were measured at least two times (*n* = 179 with 441 observations, mean 2.5 observations per woman) yielded similar results with two exceptions (Tables S9 and S10): (1) pregnancy was now significantly associated with greater odds of dehydration (OR = 1.59 [1.11–2.27; *p* = 0.011]) and (2) the odds of dehydration were only higher in 2023.

## 4 Discussion

This study examined how short‐ and long‐term water stress are associated with hydration status among Daasanach women across pregnancy, lactation, and nonreproductive states. Our results demonstrated that environmental heat stress significantly impacts hydration status among Daasanach women, regardless of reproductive status. Further, lactating women, but not pregnant women, had significantly greater odds of being dehydrated than NP/NL women. In addition to ambient temperature, higher humidity was also associated with worse hydration status in adjusted models. This finding is important because humidity can reduce the efficiency of evaporative cooling through sweating, potentially increasing physiological strain and water loss under hot conditions. This study also demonstrated that a long‐term water stress event, a historic drought, was associated with changes in the probability of dehydration. Predicted probabilities from the interaction model suggest that pregnant women experienced a sharper increase in dehydration risk at higher ambient temperatures. Lactating women, in turn, had elevated dehydration probability across much of the observed temperature range, consistent with higher baseline physiological water demands during breastfeeding. Together, these findings highlight the influence of environmental heat stress on hydration among women across reproductive states and that severe drought can further exacerbate water needs.

In our sample, pregnancy status was not significantly associated with higher odds of dehydration in the main analysis. However, when interacted with heat stress, and in the second robustness analysis, pregnancy was associated with higher odds of dehydration. This suggests that reproductive status modifies women's vulnerability to short term heat stress with pregnant women showing sharper increases in predicted probability of dehydration at higher ambient temperatures. Previous findings among Daasanach women and Tsimane’ horticulturist women in lowland Bolivia did not find that pregnancy status was associated with elevated USG, but those did not examine an interaction between heat stress and pregnancy status (Bethancourt et al. 2021). While not significant, there were signs of slightly elevated USG in late pregnancy compared to post‐first month of pregnancy (Figure S2B), which aligns with well‐documented increases in water needs while pregnant, particularly in the third trimester (Samuels et al. 2022; Food and Nutrition Board of the Institute of Medicine 2004; Yamada et al. 2022). These increased needs may be managed through behavioral adaptations. In neighboring Turkana pastoralists, for example, reproductive seasonality patterns reduced the likelihood of late pregnancy coinciding with periods of peak heat or resource scarcity (Leslie and Fry 1989; Pike 2000). Additionally, pregnancy among Daasanach women is associated with increased water borrowing, reduced engagement in physically demanding labor such as water collection, and decreases in overall physical activity particularly among women in more peripherally located communities (Sadhir et al. 2025). These social and behavioral strategies may help mitigate some of the risk of dehydration during pregnancy, even in a hot‐arid and water insecure context.

Our finding that lactating women had more concentrated urine aligns with the fact that breastfeeding further increases water needs beyond heightened needs in pregnancy and represents the most water intensive physiological state, driven by the fluid demands of breastmilk production (Howells et al. 2025). Lactating women had elevated dehydration probability across a range of observed temperatures. Lactation status in Daasanach women was significantly associated with higher urine concentration and increased odds of dehydration with greater heat stress. This parallels previous studies among Daasanach women and Tsimane’ women where lactation status was a significant predictor of dehydration and increased USG (Rosinger 2015; Bethancourt et al. 2021). Among Daasanach women, our prior work suggests that the behavioral and social accommodations made during pregnancy may not consistently extend through lactation, as lactating women continue to perform physically demanding tasks such as water collection and childcare while breastfeeding frequently (Sadhir et al. 2025). These results highlight lactation as a period of heightened vulnerability to heat stress, underscoring the need for hydration interventions tailored to the specific physiological and social demands of breastfeeding in hot, water scarce environments.

Dehydration risk was elevated for all women during the drought years of 2022–2023 and returned closer to baseline in the post‐drought year. Pregnant and lactating women faced greater dehydration risk across all years, with predicted probabilities suggesting that lactating women consistently experienced the highest dehydration risk during both drought and non‐drought years. These findings were despite the fact that 2024, the post‐drought year, recorded higher temperatures and WBGT during the study period than the drought years (2022 and 2023). This suggests that drought‐period conditions were associated with higher dehydration probability, independent of measured acute heat exposure. Behavioral accommodations made to protect pregnant women's hydration status may be difficult to sustain during prolonged drought. Pregnant Daasanach women reported less water collection, less physical activity, and more water sharing, especially during the dry season, which may contribute to better hydration outcomes (Sadhir et al. 2025). During heat stress and separately drought, however, these strategies may be less available. Increased heat stress may negate any potential coping strategies while water borrowing would be more difficult when water is scarce across households, and reducing physical activity would be more difficult to sustain with narrowed options for water sources. Focus group discussions in 2024 among Daasanach found that during drought, women were more likely to hide water they had fetched so that they would not have to share it with neighbors (Beresford et al. 2025).

The implications of the present study suggest that to fully understand women's water needs and risk of dehydration, it is essential to evaluate how short‐term factors (e.g., ambient heat stress) interact with reproductive states as well as women's water access more broadly (e.g., drought exposure). Drought related constraints on water access and evaporative cooling may have elevated dehydration risk beyond the predicted impact of increased temperature alone. This aligns with broader regional evidence of drought altering vulnerability to dehydration through water scarcity and intensifying barriers to meeting daily water needs, especially for women (Moyo et al. 2023). Daasanach communities have previously been reported to experience high levels of water insecurity (Bethancourt et al. 2023), with the 2022 historic drought further exacerbating water insecurity and chronic stress (Rosinger et al. 2024; Rosinger et al., n.d.). Exposure to drought intensifies difficulties meeting water needs for women due to increasingly scarce water sources, increasing the greater distances traveled to collect water, and heightening heat exposure during water collection. For those experiencing pregnancy or lactation, elevated physiological water needs and these pressures compound dehydration risk. These constraints have direct consequences by compounding the health risks of heat and water scarcity during drought.

### 4.1 Strengths and Limitations

This study offers some of the first insights into how environmental heat stress, hydration, and reproductive status vary across time using panel data among Daasanach women. Studying this relationship over the course of a historic drought provides a unique natural experiment to evaluate how an extreme weather event can affect risk of dehydration for this important group.

This study is also subject to some limitations. Spot urine samples do not necessarily reflect long‐term hydration status as USG can respond to recent water inputs and outputs and is delayed by approximately 30 min when compared with plasma osmolality as an indicator of hydration (Oppliger et al. 2005). USG from spot urine samples, however, remains one of the most widely used hydration biomarkers in field research due to its low cost and minimal invasiveness in resource limited settings. Further, repeat samples increase validity of results. In this study, WBGT data were available only from 2022 onward, restricting full comparisons for this heat stress measure across pre‐drought and early drought years. Finally, recording ambient heat stress at the time of urine sample collection does not capture prior heat stress events, like hot nights which likely also contribute to dehydration risk. Nevertheless, recording heat stress at the site of data collection is a strength by capturing highly acute and current microclimatic heat stress experience unlike studies that use weather records which have less resolution.

## 5 Conclusion

Extreme heat and drought compound the physiological demands of reproduction by increasing heat stress and dehydration for Daasanach semi‐nomadic pastoralist women. This study demonstrated that ambient heat stress significantly influences hydration status among Daasanach women, regardless of reproductive status. Further, lactation emerged as a consistent predictor of increased dehydration risk. Interaction models additionally suggested that reproductive status modifies women's vulnerability to short‐term heat stress with pregnant women showing sharper increases in predicted probability of dehydration at higher ambient temperatures, while lactating women had elevated dehydration probability across a range of observed temperatures. Social and behavioral accommodations made for pregnant women may mediate their risk of dehydration, yet lactating women remain highly vulnerable with elevated water needs during their entire breastfeeding period. The increased probability of dehydration observed during the 2022–2023 drought years independent of heat stress suggests that constraints on water access during drought may increase dehydration risk beyond the effects of short‐term heat exposure alone, exacerbating physiological strain in already at‐risk groups. These findings demonstrate a need for public health policy and hydration and cooling interventions that address the gendered dimensions of exposures to extreme weather events, like drought or heatwaves. Our findings underscore the need for targeted hydration and cooling interventions for women in heat‐stressed and water scarce environments and contribute to a growing body of evidence on gendered vulnerability to extreme heat stress.

## Author Contributions

**Suha Arshad:** conceptualization (equal); formal analysis (equal); writing – original draft preparation (lead); data collection (supporting); writing – review and editing (equal). **Kedir T. Roba:** conceptualization (supporting); data collection (supporting); writing – review and editing (equal). **Natalie Meriwether**, **Hannah Jacobson**, **Amanda McGrosky**, **Anna Tavormina, Grace Khosi:** data collection (supporting); writing – review and editing (supporting). **Nicole Bobbie:** methodology (supporting). **Matthew Douglass**, **David R. Braun:** funding acquisition (supporting); writing – review and editing (supporting). **Rosemary Nzunza**, **Emmanuel Ndiema:** funding acquisition, methodology (supporting), writing – review and editing (supporting). **Herman Pontzer:** funding acquisition (equal), methodology (supporting), data collection (supporting); writing – review and editing (supporting). **Asher Y. Rosinger:** conceptualization (equal); funding acquisition (lead); formal analysis (lead); writing – original draft preparation (supporting); data collection (lead); writing – review and editing (lead).

## Funding

This work was supported by the National Science Foundation (1924322, 1852406), the National Institute of Environmental Health Sciences (R01ES035402), and the Eunice Kennedy Shriver National Institute of Child Health and Human Development (P2CHD041025).

## Ethics Statement

All data were collected in accordance with procedures approved by the Pennsylvania State University's Institutional Review Board (STUDY00009589), the Kenya Medical Research Institute (KEMRI/SERU/CVR/003/3739), the Marsabit County Director of Health, and Daasanach community leaders, while Daasanach participants provided oral and written informed consent.

## Conflicts of Interest

The authors declare no conflicts of interest.

## Supporting information


**Figure S1:** Fractional polynomial fit showing ambient temperature at the military time urine samples were collected.
**Figure S2:** Association between urine specific gravity and (A) months lactating and (B) months pregnant among Daasanach women.
**Figure S3:** Association between hydration status (USG) and Wet Bulb Globe Temperature (WBGT) among Daasanach women by reproductive status.
**Figure S4:** Sensitivity analysis of predicted probability of dehydration by ambient temperature across reproductive states among reproductive‐aged women aged 16–51.
**Table S1:** Predicted probability of dehydration in Daasanach women across pre‐drought, drought, and post‐drought periods.
**Table S2:** Predicted probability of dehydration in Daasanach women across pre‐drought, drought, and post‐drought periods by reproductive status.
**Table S3:** Mixed effect linear regression nested within year and community for hydration status, urine specific gravity, as a continuous variable by ambient temperature (2019–2024) for Daasanach women.
**Table S4:** Mixed effect linear regression model testing USG including interaction between heat stress and reproductive status for Daasanach women between 2019–2024.
**Table S5:** Sensitivity analysis mixed effect logistic regression nested within year and community for odds of being dehydrated USG > 1.020 (dichotomous) by WBGT (2022–2024) for Daasanach women.
**Table S6:** Mixed effect logistic regression model testing odds of being dehydrated including interaction between WBGT and reproductive status for Daasanach women between 2019–2024.
**Table S7:** Mixed effect logistic regression model testing odds of being dehydrated for Daasanach women between 2019–2024 restricting to reproductive aged women 16–51.
**Table S8:** Mixed effect logistic regression model testing odds of being dehydrated including interaction between heat stress and reproductive status for Daasanach women between 2019–2024 restricting to reproductive aged women 16–51.
**Table S9:** Mixed effect logistic regression model testing odds of being dehydrated for Daasanach women between 2019–2024 restricting to women who participated in two or more surveys.
**Table S10:** Mixed effect logistic regression model testing odds of being dehydrated including interaction between heat stress and reproductive status for Daasanach women between 2019–2024 restricting to women who participated in two or more surveys.

## Data Availability

The data that support the findings of this study are available on request from the corresponding author. The data are not publicly available due to privacy or ethical restrictions.

## References

[ajhb70303-bib-0001] Baker, L. , H. Jacobson , A. McGrosky , et al. 2025. “Ambient Temperature and Wet Bulb Globe Temperature Outperform Heat Index in Predicting Hydration Status and Heat Perception in a Semi‐Arid Environment.” Annals of Human Biology 52, no. 1: 2456152. 10.1080/03014460.2025.2456152. 39992300 PMC11869389

[ajhb70303-bib-0002] Beresford, M. , E. A. Adams , J. Budds , et al. 2025. “Can Household Water Sharing Advance Water Security? An Integrative Review of Water Entitlements and Entitlement Failures.” Environmental Research Letters 20: 013003. 41852365 10.1088/1748-9326/ad9851 PMC12994109

[ajhb70303-bib-0003] Bethancourt, H. J. , Z. S. Swanson , R. Nzunza , et al. 2021. “Hydration in Relation to Water Insecurity, Heat Index, and Lactation Status in Two Small‐Scale Populations in Hot‐Humid and Hot‐Arid Environments.” American Journal of Human Biology: The Official Journal of the Human Biology Council 33, no. 1: e23447. 10.1002/ajhb.23447. 32583580 PMC8829588

[ajhb70303-bib-0004] Bethancourt, H. J. , Z. S. Swanson , R. Nzunza , et al. 2023. “The Co‐Occurrence of Water Insecurity and Food Insecurity Among Daasanach Pastoralists in Northern Kenya.” Public Health Nutrition 26, no. 3: 693–703. 10.1017/S1368980022001689. 35941080 PMC9989708

[ajhb70303-bib-0005] Bhandari, D. , E. Robinson , W. Pollock , J. Watterson , T. T. Su , and Z. Lokmic‐Tomkins . 2025. “Mapping Multilevel Adaptation Response to Protect Maternal and Child Health From Climate Change Impacts: A Scoping Review.” iScience 28, no. 3: 111914. 10.1016/j.isci.2025.111914. 40092619 PMC11907458

[ajhb70303-bib-0006] Bonell, A. , J. Hirst , A. M. Vicedo‐Cabrera , A. Haines , A. M. Prentice , and N. S. Maxwell . 2020. “A Protocol for an Observational Cohort Study of Heat Strain and Its Effect on Fetal Wellbeing in Pregnant Farmers in The Gambia.” Wellcome Open Research 5: 32. 10.12688/wellcomeopenres.15731.2. 32292825 PMC7141168

[ajhb70303-bib-0007] Chersich, M. F. , M. D. Pham , A. Areal , et al. 2020. “Associations Between High Temperatures in Pregnancy and Risk of Preterm Birth, Low Birth Weight, and Stillbirths: Systematic Review and Meta‐Analysis.” BMJ 271: m3811. 10.1136/bmj.m3811. PMC7610201 33148618

[ajhb70303-bib-0008] Cheung, K. L. , and R. A. Lafayette . 2013. “Renal Physiology of Pregnancy.” Advances in Chronic Kidney Disease 20, no. 3: 209–214. 10.1053/j.ackd.2013.01.012. 23928384 PMC4089195

[ajhb70303-bib-0009] Costantine, M. M. 2014. “Physiologic and Pharmacokinetic Changes in Pregnancy.” Frontiers in Pharmacology 5: 65. 10.3389/fphar.2014.00065. 24772083 PMC3982119

[ajhb70303-bib-0010] Ebi, K. L. , A. Capon , P. Berry , et al. 2021. “Hot Weather and Heat Extremes: Health Risks.” Lancet 398, no. 10301: 698–708. 10.1016/S0140-6736(21)01208-3. 34419205

[ajhb70303-bib-0012] Food and Nutrition Board of the Institute of Medicine . 2004. Dietary Reference Intakes for Water, Potassium, Sodium, Chloride, and Sulfate. National Academic Press.

[ajhb70303-bib-0013] Ford, L. B. , H. J. Bethancourt , Z. S. Swanson , et al. 2023. “Water Insecurity, Water Borrowing and Psychosocial Stress Among Daasanach Pastoralists in Northern Kenya.” Water International 48, no. 1: 63–86. 10.1080/02508060.2022.2138050. 38800511 PMC11126231

[ajhb70303-bib-0014] Hew‐Butler, T. D. , C. Eskin , J. Bickham , M. Rusnak , and M. VanderMeulen . 2018. “Dehydration Is How You Define It: Comparison of 318 Blood and Urine Athlete Spot Checks.” BMJ Open Sport & Exercise Medicine 4, no. 1: e000297. 10.1136/bmjsem-2017-000297. PMC5812394 29464103

[ajhb70303-bib-0015] Howells, M. , A. E. L. Palmquist , C. Josefson , et al. 2025. “Climate Change, Evolution, and Reproductive Health: The Impact of Water Insecurity and Heat Stress on Pregnancy and Lactation.” Evolution, Medicine, and Public Health 13, no. 1: 125–139. 10.1093/emph/eoaf008. 40574887 PMC12199371

[ajhb70303-bib-0016] Kenya National Bureau of Statistics (KNBS) . 2019. Ethnic Affiliation. Census. Kenya National Bureau of Statistics (KNBS).

[ajhb70303-bib-0017] Kim, J. , A. Lee , and M. Rossin‐Slater . 2019. What to Expect When It Gets Hotter: The Impacts of Prenatal Exposure to Extreme Heat on Maternal Health (No. w26384; p. w26384). National Bureau of Economic Research. 10.3386/w26384.

[ajhb70303-bib-0018] Kim, S. Y. , and D. Y. Yi . 2020. “Components of Human Breast Milk: From Macronutrient to Microbiome and microRNA.” Clinical and Experimental Pediatrics 63, no. 8: 301–309. 10.3345/cep.2020.00059. 32252145 PMC7402982

[ajhb70303-bib-0019] Leslie, P. W. , and P. H. Fry . 1989. “Extreme Seasonality of Births Among Nomadic Turkana Pastoralists.” American Journal of Physical Anthropology 79, no. 1: 103–115. 10.1002/ajpa.1330790111. 2750875

[ajhb70303-bib-0020] Li, S. , X. Xiao , and X. Zhang . 2023. “Hydration Status in Older Adults: Current Knowledge and Future Challenges.” Nutrients 15, no. 11: 2609. 10.3390/nu15112609. 37299572 PMC10255140

[ajhb70303-bib-0021] Luber, G. , and M. McGeehin . 2008. “Climate Change and Extreme Heat Events.” American Journal of Preventive Medicine 35, no. 5: 429–435. 10.1016/j.amepre.2008.08.021. 18929969

[ajhb70303-bib-0022] Mao, Y. , Q. Gao , Y. Zhang , et al. 2023. “Associations Between Extreme Temperature Exposure and Hypertensive Disorders in Pregnancy: A Systematic Review and Meta‐Analysis.” Hypertension in Pregnancy 42, no. 1: 2288586. 10.1080/10641955.2023.2288586. 38053322

[ajhb70303-bib-0023] McGrosky, A. , L. Ford , E. Hinz , et al. 2025. “High Water Turnover, Hydration Status, and Heat Stress Among Daasanach Pastoralists in a Hot, Semi‐Arid Climate.” Evolution, Medicine, and Public Health 13, no. 1: 215–228. 40917635 10.1093/emph/eoaf017 PMC12409784

[ajhb70303-bib-0024] McGrosky, A. , Z. S. Swanson , R. Rimbach , et al. 2024. “Total Daily Energy Expenditure and Elevated Water Turnover in a Small‐Scale Semi‐Nomadic Pastoralist Society From Northern Kenya.” Annals of Human Biology 51, no. 1: 2310724. 10.1080/03014460.2024.2310724. 38594936 PMC11567135

[ajhb70303-bib-0025] Moyo, E. , L. G. Nhari , P. Moyo , G. Murewanhema , and T. Dzinamarira . 2023. “Health Effects of Climate Change in Africa: A Call for an Improved Implementation of Prevention Measures.” Eco‐Environment & Health 2, no. 2: 74–78. 10.1016/j.eehl.2023.04.004. 38075293 PMC10702879

[ajhb70303-bib-0026] Muller, C. J. , and R. F. MacLehose . 2014. “Estimating Predicted Probabilities From Logistic Regression: Different Methods Correspond to Different Target Populations.” International Journal of Epidemiology 43, no. 3: 962–970. 24603316 10.1093/ije/dyu029 PMC4052139

[ajhb70303-bib-0027] Mwamidi, D. , J. G. Renom , A. Fernandez‐Llamazares Onrubia , D. Burgas Riera , P. Domínguez , and M. D. M. Cabeza‐Jaimejuan . 2018. “Contemporary Pastoral Commons in East Africa as OECMs: A Case Study From the Daasanach Community.” Parks 24: 79–88. 10.2305/IUCN.CH.2018.PARKS-24-SIDMM.en.

[ajhb70303-bib-0028] Neville, M. C. , R. Keller , J. Seacat , et al. 1988. “Studies in Human Lactation: Milk Volumes in Lactating Women During the Onset of Lactation and Full Lactation.” American Journal of Clinical Nutrition 48, no. 6: 1375–1386. 10.1093/ajcn/48.6.1375. 3202087

[ajhb70303-bib-0029] Odongo, R. A. , T. Schrieks , I. Streefkerk , et al. 2025. “Drought Impacts and Community Adaptation: Perspectives on the 2020–2023 Drought in East Africa.” International Journal of Disaster Risk Reduction 119: 105309. 10.1016/j.ijdrr.2025.105309.

[ajhb70303-bib-0030] Ojwang, W. , K. O. Obiero , O. O. Donde , et al. 2018. “Lake Turkana: World's Largest Permanent Desert Lake (Kenya).” In The Wetland Book, edited by C. Finlayson , G. Milton , R. Prentice , and N. Davidson . Springer. 10.1007/978-94-007-4001-3_254.

[ajhb70303-bib-0031] Oppliger, R. A. , S. A. Magnes , L. R. A. Popowski , and C. V. Gisolfi . 2005. “Accuracy of Urine Specific Gravity and Osmolality as Indicators of Hydration Status.” International Journal of Sport Nutrition and Exercise Metabolism 15, no. 3: 236–251. 10.1123/ijsnem.15.3.236. 16131695

[ajhb70303-bib-0033] Pike, I. L. 2000. “Pregnancy Outcome for Nomadic Turkana Pastoralists of Kenya.” American Journal of Physical Anthropology 113, no. 1: 31–45. 10.1002/1096-8644(200009)113:1<31::AID-AJPA4>3.0.CO;2-W. 10954618

[ajhb70303-bib-0034] Roba, K. T. , H. Jacobson , A. McGrosky , et al. 2025. “Chronic Stress and Severe Water Insecurity During the Historic 2022 Drought in Northern Kenya Were Associated With Inflammation Among Daasanach Seminomadic Pastoralists.” American Journal of Human Biology 37, no. 1: e70009. 10.1002/ajhb.70009. 39916292 PMC11803130

[ajhb70303-bib-0035] Romanello, M. , M. Walawender , S. C. Hsu , et al. 2024. “The 2024 Report of the Lancet Countdown on Health and Climate Change: Facing Record‐Breaking Threats From Delayed Action.” Lancet 404, no. 10465: 1847–1896. 10.1016/S0140-6736(24)01822-1. 39488222 PMC7616816

[ajhb70303-bib-0036] Rosinger, A. 2015. “Heat and Hydration Status: Predictors of Repeated Measures of Urine Specific Gravity Among Tsimane’ Adults in the Bolivian Amazon.” American Journal of Physical Anthropology 158, no. 4: 696–707. 10.1002/ajpa.22813. 26213151

[ajhb70303-bib-0037] Rosinger, A. Y. 2023. “Extreme Climatic Events and Human Biology and Health: A Primer and Opportunities for Future Research.” American Journal of Human Biology 35, no. 1: e23843. 36449411 10.1002/ajhb.23843 PMC9840683

[ajhb70303-bib-0038] Rosinger, A. Y. , M. Douglass , A. McGrosky , et al. n.d. Historic Drought in Northern Kenya Shifts Diet and Mobility Away From Traditional Pastoralism as Resource Security and Nutrition Falter. Under Review. 10.1016/j.gloenvcha.2026.103212 PMC13484248 42614764

[ajhb70303-bib-0039] Rosinger, A. Y. , J. Stoler , L. B. Ford , et al. 2024. “Mobility Ideation due to Water Problems During Historic 2022 Drought Associated With Livestock Wealth, Water and Food Insecurity, and Fingernail Cortisol Concentration in Northern Kenya.” Social Science & Medicine 359: 117280. 10.1016/j.socscimed.2024.117280. 39236480 PMC11456390

[ajhb70303-bib-0040] Sadhir, S. , A. McGrosky , L. B. Ford , et al. 2025. “Physical Activity and Pregnancy Norms Among Daasanach Semi‐Nomadic Pastoralist Women in Northern Kenya.” American Journal of Human Biology 37, no. 1: e24174. 10.1002/ajhb.24174. 39463015 PMC11669534

[ajhb70303-bib-0041] Samuels, L. , B. Nakstad , N. Roos , et al. 2022. “Physiological Mechanisms of the Impact of Heat During Pregnancy and the Clinical Implications: Review of the Evidence From an Expert Group Meeting.” International Journal of Biometeorology 66, no. 8: 1505–1513. 10.1007/s00484-022-02301-6. 35554684 PMC9300488

[ajhb70303-bib-0043] Tofu, D. A. , T. Dilbato , C. Fana , G. Abebe , K. Hussein , and A. Mohammed . 2025. “Analysis of Vulnerability, Its Drivers, and Strategies Applied Towards Reducing the Pastoral and Agro‐Pastoral Livelihood Vulnerability to Climatic Shocks.” Scientific Reports 15: 2567. 10.1038/s41598-024-79165-w. 39833278 PMC11747342

[ajhb70303-bib-0044] Traylor, D. O. , W. Cameron , B. Clark , E. Anderson , R. Henderson , and L. Clark . 2024. “Impact of Climate Change on Human Lactation: Biological, Socioeconomic, and Public Health Implications [Version 1; Peer Review: 1 Approved, 1 Approved With Reservations, 1 Not Approved].” F1000Research 13: 993. 10.12688/f1000research.155447.1.

[ajhb70303-bib-0045] UNICEF . 2022. Advocacy Brief: Water Crisis in the Horn of Africa. https://www.unicef.org/media/126006/file/water‐crisis‐horn‐africa‐2022.pdf.

[ajhb70303-bib-0046] United Nations Children's Fund . 2023. Protecting Children From Heat Stress: A Technical Note. https://www.unicef.org/media/139926/file/Protecting‐children‐from‐heat‐stress‐A‐technical‐note‐2023.pdf.

[ajhb70303-bib-0047] Vecellio, D. J. , S. T. Wolf , R. M. Cottle , and W. L. Kenney . 2022. “Evaluating the 35°C Wet‐Bulb Temperature Adaptability Threshold for Young, Healthy Subjects (PSU HEAT Project).” Journal of Applied Physiology 132, no. 2: 340–345. 10.1152/japplphysiol.00738.2021. 34913738 PMC8799385

[ajhb70303-bib-0048] Vogel, M. M. , R. Orth , F. Cheruy , et al. 2017. “Regional Amplification of Projected Changes in Extreme Temperatures Strongly Controlled by Soil Moisture‐Temperature Feedbacks.” Geophysical Research Letters 44: 1511–1519. 10.1002/2016GL071235.

[ajhb70303-bib-0049] Vricella, L. K. 2017. “Emerging Understanding and Measurement of Plasma Volume Expansion in Pregnancy.” American Journal of Clinical Nutrition 106, no. Suppl 6: 1620S–1625S. 10.3945/ajcn.117.155903. 29070547 PMC5701717

[ajhb70303-bib-0050] Wall, U. 2020. A Visit to a Daasanach Village—Omo Valley, Ethiopia. Ursula's Weekly Wanders. 2025. https://www.ursulasweeklywanders.com/travel/a‐visit‐to‐a‐daasanach‐village‐omo‐valley‐ethiopia/#:~:text=Daasanach%20or%20Dassanech%20means%20People,extra%20pressures%20on%20food%20supply.

[ajhb70303-bib-0052] Weather and Climate . 2020. Turkana, Kenya Climate. 2025. https://weatherandclimate.com/kenya/turkana.

[ajhb70303-bib-0053] World Health Organization . 2011. Exclusive Breastfeeding for Six Months Best for Babies Everywhere. 2025. https://www.who.int/news/item/15‐01‐2011‐exclusive‐breastfeeding‐for‐six‐months‐best‐for‐babies‐everywhere.

[ajhb70303-bib-0054] World Health Organization . 2023. Climate Change and Health. 2025. https://www.who.int/news‐room/fact‐sheets/detail/climate‐change‐and‐health.

[ajhb70303-bib-0055] Wutich, A. , A. Y. Rosinger , J. Stoler , W. Jepson , and A. Brewis . 2020. “Measuring Human Water Needs.” American Journal of Human Biology 32, no. 1: e23350. 31702101 10.1002/ajhb.23350 PMC7050503

[ajhb70303-bib-0056] Yamada, Y. , X. Zhang , M. E. T. Henderson , et al. 2022. “Variation in Human Water Turnover Associated With Environmental and Lifestyle Factors.” Science 378, no. 6622: 909–915. 10.1126/science.abm8668. 36423296 PMC9764345

[ajhb70303-bib-0057] Zhou, S. , A. P. Williams , A. M. Berg , et al. 2019. “Land–Atmosphere Feedbacks Exacerbate Concurrent Soil Drought and Atmospheric Aridity.” Proceedings of the National Academy of Sciences of the United States of America 116, no. 38: 18848–18853. 10.1073/pnas.1904955116. 31481606 PMC6754607

