# Supplementary material for: Increased Vulnerability to Dehydration Due to Heat Stress and Drought Across Reproductive States for Pastoralist Women in Northern Kenya
Source: Am J Hum Biol. 2026 Jun 30;38(7):e70303. doi: 10.1002/ajhb.70303 (PMC13320144; doi:10.1002/ajhb.70303)
Supplement: Supplementary file 1 — Figure S1: Fractional polynomial fit showing ambient temperature at the military time urine samples were collected. Figure S2: Association between urine specific gravity and (A) months lactating and (B) months pregnant among Daasanach women. Figure S3: Association between hydration status (USG) and Wet Bulb Globe Temperature (WBGT) among Daasanach women by reproductive status. Figure S4: Sensitivity analysis of predicted probability of dehydration by ambient temperature across reproductive states among reproductive‐aged women aged 16–51. Table S1: Predicted probability of dehydration in Daasanach women across pre‐drought, drought, and post‐drought periods. Table S2: Predicted probability of dehydration in Daasanach women across pre‐drought, drought, and post‐drought periods by reproductive status. Table S3: Mixed effect linear regression nested within year and community for hydration status, urine specific gravity, as a continuous variable by ambient temperature (2019–2024) for Daasanach women. Table S4: Mixed effect linear regression model testing USG including interaction between heat stress and reproductive status for Daasanach women between 2019–2024. Table S5: Sensitivity analysis mixed effect logistic regression nested within year and community for odds of being dehydrated USG > 1.020 (dichotomous) by WBGT (2022–2024) for Daasanach women. Table S6: Mixed effect logistic regression model testing odds of being dehydrated including interaction between WBGT and reproductive status for Daasanach women between 2019–2024. Table S7: Mixed effect logistic regression model testing odds of being dehydrated for Daasanach women between 2019–2024 restricting to reproductive aged women 16–51. Table S8: Mixed effect logistic regression model testing odds of being dehydrated including interaction between heat stress and reproductive status for Daasanach women between 2019–2024 restricting to reproductive aged women 16–51. Table S9: Mixed effect logistic regression model testin [file AJHB-38-e70303-s001.docx]

**Supplemental materials for**

**Title**: Increased vulnerability to dehydration due to heat stress and drought across reproductive states for pastoralist women in northern Kenya

Figure S1: Fractional polynomial fit showing ambient temperature at the military time urine samples were collected

Figure S2: Association between urine specific gravity and (A) months lactating and (B) months pregnant among Daasanach women

Note: Reference line at 1.020 indicates dehydration.

Figure S3: Association between hydration status (USG) and Wet Bulb Globe Temperature (WBGT) among Daasanach women by reproductive status


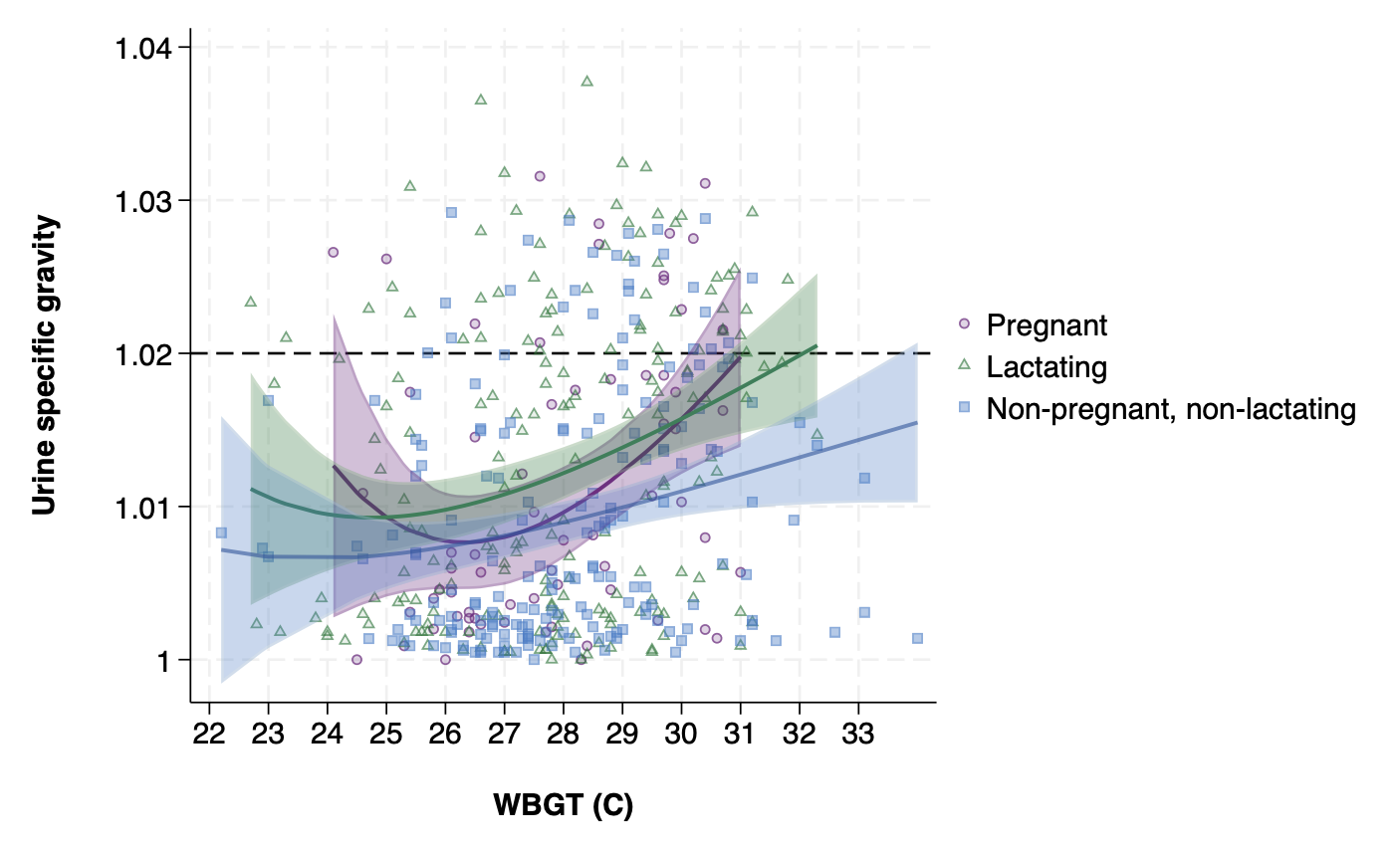


The dashed line represents the USG cutoff of 1.020 used to classify dehydration.

Figure S4. Sensitivity analysis of predicted probability of dehydration by ambient temperature across reproductive states among reproductive-aged women aged 16-51

Model generated using post-estimation marginalization to visualize interaction between heat stress and reproductive status using model presented in Table S4.Table S1: Predicted probability of dehydration in Daasanach women across pre-drought, drought, and post-drought periods

| Year | Marginal Predicted Mean | 95% Confidence Interval | p-value |
| --- | --- | --- | --- |
| 2019 | 0.177 | [0.163, 0.190] | <0.001*** |
| 2022 | 0.229 | [0.215, 0.242] | <0.001*** |
| 2023 | 0.225 | [0.208, 0.243] | <0.001*** |
| 2024 | 0.172 | [0.156, 0.187] | <0.001*** |
|  |  |  |  |

*** p<0.001 ** p<0.01 * p<0.05.

Table S2: Predicted probability of dehydration in Daasanach women across pre-drought, drought, and post-drought periods by reproductive status

| Year | Marginal Predicted Mean | 95% Confidence Interval | p-value |
| --- | --- | --- | --- |
| 2019:  Non-pregnant, non-lactating | 0.128 | [0.103, 0.152] | <0.001*** |
| Pregnant | 0.173 | [0.116, 0.230] | <0.001*** |
| Lactating | 0.217 | [0.201, 0.233] | <0.001*** |
| 2022:  Non-pregnant, non-lactating | 0.170 | [0.131, 0.209] | <0.001*** |
| Pregnant | 0.226 | [0.181, 0.271] | <0.001*** |
| Lactating | 0.277 | [0.249, 0.306] | <0.001*** |
| 2023:  Non-pregnant, non-lactating | 0.167 | [0.136, 0.199] | <0.001*** |
| Pregnant | 0.223 | [0.165, 0.281] | <0.001*** |
| Lactating | 0.274 | [0.240, 0.307] | <0.001*** |
| 2024:  Non-pregnant, non-lactating | 0.123 | [0.095, 0.152] | <0.001*** |
| Pregnant | 0.168 | [0.122, 0.215] | <0.001*** |
| Lactating | 0.211 | [0.186, 0.236] | <0.001*** |
|  |  |  |  |

*** p<0.001 ** p<0.01 * p<0.05.

Table S3: Mixed effect linear regression nested within year and community for hydration status, urine specific gravity, as a continuous variable by ambient temperature (2019-2024) for Daasanach women.

| Variable | Coefficient (USG) | 95% CI | p-value |
| --- | --- | --- | --- |
| Ambient Temp (°C) | 0.0015 | [0.0012, 0.0018] | <0.001*** |
| Humidity (%) | 2.20e-04 | [3.34e-05, 4.06e-04] | 0.021* |
| Non-pregnant, non-lactating | Reference | - | - |
| Pregnant | 0.0016 | [-9.12e-04, 0.0040] | 0.216 |
| Lactating | 0.0027 | [0.0012, 0.0042] | <0.001*** |
| Body fat % | 3.87e-05 | [-1.42e-04, 2.19e-04] | 0.675 |
| Age | -5.63e-05 | [-9.69e-05, -1.56e-05] | 0.007** |
| Household size | -5.10e-04 | [-7.71e-04, -2.49e-04] | <0.001*** |
| Year: 2019 (pre-drought) | Reference | - | - |
| 2022 (peak drought) | 0.0018 | [6.71e-05, 0.0036] | 0.042* |
| 2023 (end of drought) | 0.0016 | [8.50e-05, 0.0030] | 0.038* |
| 2024 (1-year post-drought) | 3.70e-04 | [-8.43e-04, 0.0016] | 0.550 |
| Constant | 0.9544 | [0.9380, 0.9707] | <0.001*** |
| Observations | 565 |  |  |
| N | 303 women |  |  |

*** p<0.001 ** p<0.01 * p<0.05. CI: confidence interval. Mixed effect model nested observations within year and community residence.

Table S4: Mixed effect linear regression model testing USG including interaction between heat stress and reproductive status for Daasanach women between 2019-2024

| Variable | Coefficient (USG) | 95% CI | p-value |
| --- | --- | --- | --- |
| Ambient Temperature (°C) | 8.34e-04 | [-7.35e-04, 0.0024] | 0.297 |
| Humidity (%) | -8.92e-05 | [-0.0011, 9.13e-04] | 0.861 |
| Temp × Humidity | 9.49e-06 | [-2.76e-05, 4.66e-05] | 0.616 |
| Non-pregnant, non-lactating | Reference | - | - |
| Pregnant | -0.1988 | [-0.3083, -0.0894] | <0.001*** |
| Lactating | -0.0332 | [-0.0867, 0.0203] | 0.224 |
| Pregnant × Temp | 0.0059 | [0.0027, 0.0090] | <0.001*** |
| Lactating × Temp | 0.0011 | [-3.15e-04, 0.0026] | 0.126 |
| Pregnant × Humidity | 0.0040 | [0.0020, 0.0061] | <0.001*** |
| Lactating × Humidity | 6.27e-04 | [-5.96e-05, 0.0013] | 0.073 |
| Pregnant × Temp × Humidity | -1.18e-04 | [-1.81e-04, -5.44e-05] | <0.001*** |
| Lactating × Temp × Humidity | -2.00e-05 | [-3.90e-05, -9.48e-07] | 0.040* |
| Body fat % | 3.01e-05 | [-1.46e-04, 2.06e-04] | 0.737 |
| Age | -6.04e-05 | [-1.06e-04, -1.53e-05] | 0.009** |
| Household size | -5.02e-04 | [-8.09e-04, -1.94e-04] | 0.001** |
| Year: 2019 (pre-drought) | Reference | - | - |
| 2022 (peak drought) | 0.0020 | [3.85e-04, 0.0036] | 0.015* |
| 2023 (end of drought) | 0.0017 | [4.82e-04, 0.0030] | 0.007** |
| 2024 (1-year post-drought) | 7.00e-04 | [-4.31e-04, 0.0018] | 0.225 |
| Constant | 0.9771 | [0.9260, 1.0282] | <0.001*** |
|  |  |  |  |
| Observations | 565 |  |  |
| N | 303 women |  |  |

*** p<0.001 ** p<0.01 * p<0.05. CI: confidence interval. Mixed effect model nested observations within year and community residence. Three-way interaction includes all main terms and two-way interactions.

Table S5: Sensitivity analysis mixed effect logistic regression nested within year and community for odds of being dehydrated USG>1.020 (dichotomous) by WBGT (2022-2024) for Daasanach women

| Variable | Odds Ratio (USG>1.020) | 95% CI | p-value |
| --- | --- | --- | --- |
| WBGT (°C) | 1.26 | [1.21, 1.31] | <0.001*** |
| Non-pregnant, non-lactating | Reference | - | - |
| Pregnant | 1.24 | [0.61, 2.54] | 0.554 |
| Lactating | 1.96 | [1.22, 3.14] | 0.005** |
| Body fat % | 1.00 | [0.95, 1.05] | 0.908 |
| Age | 0.97 | [0.96, 0.99] | <0.001*** |
| Household size | 0.89 | [0.80, 0.98] | 0.024* |
| Year: 2022 (peak drought) | Reference | - | - |
| 2023 (end of drought) | 1.01 | [0.96, 1.08] | 0.637 |
| 2024 (1-year post-drought) | 0.76 | [0.60, 0.97] | 0.025* |
| Constant | 0.00 | [1.63e-04, 0.01] | <0.001*** |
|  |  |  |  |
| Observations | 446 |  |  |
| N | 272 women |  |  |

*** p<0.001 ** p<0.01 * p<0.05. CI: confidence interval. Mixed effect model nested observations within year and community residence.

Table S6: Mixed effect logistic regression model testing odds of being dehydrated including interaction between WBGT and reproductive status for Daasanach women between 2019-2024

| Variable | Odds Ratio  (USG>1.020) | 95% CI | p-value |
| --- | --- | --- | --- |
| WBGT (°C) | 1.27 | [1.00, 1.61] | 0.049* |
| Non-pregnant, non-lactating | Reference | - | - |
| Pregnant | 0.19 | [2.70e-08, 1.29e+06] | 0.834 |
| Lactating | 4.48 | [1.04e-04, 1.94e+05] | 0.783 |
| Pregnant × WBGT | 1.07 | [0.63, 1.81] | 0.802 |
| Lactating × WBGT | 0.97 | [0.67, 1.40] | 0.875 |
| Body fat % | 1.00 | [0.95, 1.05] | 0.907 |
| Age | 0.97 | [0.96, 0.99] | 0.001** |
| Household size | 0.88 | [0.79, 0.99] | 0.034* |
| Year: 2022 (peak drought) | Reference | - | - |
| 2023 (end of drought) | 1.01 | [0.92, 1.11] | 0.887 |
| 2024 (1-year post-drought) | 0.76 | [0.60, 0.97] | 0.027* |
| Constant | 0.00 | [7.96e-07, 1.82] | 0.072 |
|  |  |  |  |
| Observations | 446 |  |  |
| N | 272 women |  |  |

*** p<0.001 ** p<0.01 * p<0.05. CI: confidence interval. WBGT: Wet bulb globe temperature. Mixed effect model nested observations within year and community residence. Three-way interaction includes all main terms and two-way interactions.

Table S7: Mixed effect logistic regression model testing odds of being dehydrated for Daasanach women between 2019-2024 restricting to reproductive aged women 16-51

| Variable | Odds Ratio  (USG>1.020) | 95% CI | p-value |
| --- | --- | --- | --- |
| Ambient Temp (°C) | 1.39 | [1.30, 1.49] | <0.001*** |
| Humidity (%) | 1.03 | [1.02, 1.04] | <0.001*** |
| Non-pregnant, non-lactating | Reference | - | - |
| Pregnant | 1.33 | [0.74, 2.37] | 0.338 |
| Lactating | 1.82 | [1.22, 2.72] | 0.003** |
| Body fat % | 1.02 | [0.98, 1.06] | 0.356 |
| Age | 0.95 | [0.93, 0.97] | <0.001*** |
| Household size | 0.93 | [0.85, 1.02] | 0.142 |
| Year: 2019 (pre-drought) | Reference | - | - |
| 2022 (peak drought) | 1.27 | [1.11, 1.45] | 0.001** |
| 2023 (end of drought) | 1.42 | [1.30, 1.54] | <0.001*** |
| 2024 (1-year post-drought) | 0.96 | [0.75, 1.22] | 0.722 |
| Constant | 3.44e-06 | [1.72e-07, 6.89e-05] | <0.001*** |
|  |  |  |  |
| Observations | 496 |  |  |
| N | 276 women |  |  |

*** p<0.001 ** p<0.01 * p<0.05. CI: confidence interval. Mixed effect model nested observations within year and community residence.

Table S8: Mixed effect logistic regression model testing odds of being dehydrated including interaction between heat stress and reproductive status for Daasanach women between 2019-2024 restricting to reproductive aged women 16-51

| Variable | Odds Ratio (USG>1.020) | 95% CI | p-value |
| --- | --- | --- | --- |
| Ambient Temperature (°C) | 0.64 | [0.20, 2.07] | 0.453 |
| Humidity (%) | Reference | - | - |
| Temp × Humidity | 1.52e-23 | [1.55e-34, 1.48e-12] | <0.001*** |
| Non-pregnant, non-lactating | 2.52e-06 | [2.18e-20, 2.92e+08] | 0.435 |
| Pregnant | 4.69 | [2.29, 9.59] | <0.001*** |
| Lactating | 1.55 | [0.63, 3.81] | 0.342 |
| Pregnant × Temp | 0.49 | [0.19, 1.26] | 0.138 |
| Lactating × Temp | 1.02 | [1.00, 1.05] | 0.103 |
| Pregnant × Humidity | 3.61 | [1.56, 8.37] | 0.003** |
| Lactating × Humidity | 1.62 | [0.76, 3.47] | 0.214 |
| Pregnant × Temp × Humidity | 0.96 | [0.94, 0.99] | 0.003** |
| Lactating × Temp × Humidity | 0.98 | [0.96, 1.01] | 0.158 |
| Body fat % | 1.02 | [0.98, 1.07] | 0.366 |
| Age | 0.95 | [0.93, 0.97] | <0.001*** |
| Household size | 0.93 | [0.82, 1.06] | 0.311 |
| Year: 2019 (pre-drought) | Reference | - | - |
| 2022 (peak drought) | 1.37 | [1.09, 1.71] | 0.007** |
| 2023 (end of drought) | 1.44 | [1.14, 1.83] | 0.002** |
| 2024 (1-year post-drought) | 0.96 | [0.76, 1.21] | 0.722 |
| Constant | 4.42e+05 | [3.11e-13, 6.29e+23] | 0.542 |
|  |  |  |  |
| Observations | 496 |  |  |
| N | 276 women |  |  |

*** p<0.001 ** p<0.01 * p<0.05. CI: confidence interval. Mixed effect model nested observations within year and community residence. Three-way interaction includes all main terms and two-way interactions.

Table S9: Mixed effect logistic regression model testing odds of being dehydrated for Daasanach women between 2019-2024 restricting to women who participated in two or more surveys

| Variable | Odds Ratio  (USG>1.020) | 95% CI | p-value |
| --- | --- | --- | --- |
| Ambient Temp (°C) | 1.40 | [1.28, 1.52] | <0.001*** |
| Humidity (%) | 1.03 | [1.02, 1.05] | <0.001*** |
| Non-pregnant, non-lactating | Reference | - | - |
| Pregnant | 1.59 | [1.11, 2.27] | 0.011* |
| Lactating | 2.54 | [2.17, 2.98] | <0.001*** |
| Body fat % | 1.02 | [0.96, 1.09] | 0.500 |
| Age | 0.98 | [0.95, 1.00] | 0.063 |
| Household size | 0.87 | [0.79, 0.97] | 0.008** |
| Year: 2019 (pre-drought) | Reference | - | - |
| 2022 (peak drought) | 1.09 | [0.98, 1.21] | 0.111 |
| 2023 (end of drought) | 1.06 | [1.01, 1.10] | 0.013* |
| 2024 (1-year post-drought) | 0.74 | [0.64, 0.85] | <0.001*** |
| Constant | 2.04e-06 | [1.53e-07, 2.72e-05] | <0.001*** |
|  |  |  |  |
| Observations | 441 |  |  |
| N | 179 women |  |  |

*** p<0.001 ** p<0.01 * p<0.05. CI: confidence interval. Mixed effect model nested observations within year and community residence.

Table S10: Mixed effect logistic regression model testing odds of being dehydrated including interaction between heat stress and reproductive status for Daasanach women between 2019-2024 restricting to women who participated in two or more surveys

| Variable | Odds Ratio  (USG>1.020) | 95% CI | p-value |
| --- | --- | --- | --- |
| Ambient Temperature (°C) | 0.44 | [0.11, 1.78] | 0.251 |
| Humidity (%) | 0.37 | [0.11, 1.19] | 0.096 |
| Temp × Humidity | 1.03 | [1.00, 1.07] | 0.073 |
| Non-pregnant, non-lactating | Reference | - | - |
| Pregnant | 5.02e-28 | [1.50e-49, 1.68e-06] | 0.013* |
| Lactating | 1.50e-10 | [1.12e-36, 2.01e+16] | 0.461 |
| Pregnant × Temp | 6.13 | [1.63, 23.09] | 0.007** |
| Lactating × Temp | 1.98 | [0.37, 10.58] | 0.424 |
| Pregnant × Humidity | 4.24 | [1.30, 13.89] | 0.017* |
| Lactating × Humidity | 1.88 | [0.45, 7.83] | 0.385 |
| Pregnant × Temp × Humidity | 0.96 | [0.93, 0.99] | 0.012* |
| Lactating × Temp × Humidity | 0.98 | [0.94, 1.02] | 0.363 |
| Body fat % | 1.02 | [0.97, 1.08] | 0.423 |
| Age | 0.97 | [0.95, 1.00] | 0.047* |
| Household size | 0.87 | [0.74, 1.02] | 0.094 |
| Year: 2019 (pre-drought) | Reference | - | - |
| 2022 (peak drought) | 1.16 | [0.98, 1.36] | 0.077 |
| 2023 (end of drought) | 1.04 | [0.75, 1.44] | 0.817 |
| 2024 (1-year post-drought) | 0.73 | [0.56, 0.97] | 0.028* |
| Constant | 2.09e+11 | [3.38e-11, 1.29e+33] | 0.309 |
|  |  |  |  |
| Observations | 441 |  |  |
| N | 179 women |  |  |

*** p<0.001 ** p<0.01 * p<0.05. CI: confidence interval. Mixed effect model nested observations within year and community residence. Three-way interaction includes all main terms and two-way interactions.
